# Histone ChIP‐Seq identifies differential enhancer usage during chondrogenesis as critical for defining cell‐type specificity

**DOI:** 10.1096/fj.201902061RR

**Published:** 2020-02-14

**Authors:** Kathleen Cheung, Matthew J. Barter, Julia Falk, Carole J. Proctor, Louise N. Reynard, David A. Young

**Affiliations:** ^1^ Skeletal Research Group Institute of Genetic Medicine Newcastle University Central Parkway Newcastle upon Tyne UK; ^2^ Bioinformatics Support Unit Faculty of Medical Sciences Newcastle University Newcastle upon Tyne UK

**Keywords:** cartilage, chondrocyte development, chromatin

## Abstract

Epigenetic mechanisms are known to regulate gene expression during chondrogenesis. In this study, we have characterized the epigenome during the in vitro differentiation of human mesenchymal stem cells (hMSCs) into chondrocytes. Chromatin immunoprecipitation followed by next‐generation sequencing (ChIP‐seq) was used to assess a range of N‐terminal posttranscriptional modifications (marks) to histone H3 lysines (H3K4me3, H3K4me1, H3K27ac, H3K27me3, and H3K36me3) in both hMSCs and differentiated chondrocytes. Chromatin states were characterized using histone ChIP‐seq and *cis*‐regulatory elements were identified in chondrocytes. Chondrocyte enhancers were associated with chondrogenesis‐related gene ontology (GO) terms. In silico analysis and integration of DNA methylation data with chondrogenesis chromatin states revealed that enhancers marked by histone marks H3K4me1 and H3K27ac were de‐methylated during in vitro chondrogenesis. Similarity analysis between hMSC and chondrocyte chromatin states defined in this study with epigenomes of cell‐types defined by the Roadmap Epigenomics project revealed that enhancers are more distinct between cell‐types compared to other chromatin states. Motif analysis revealed that the transcription factor SOX9 is enriched in chondrocyte enhancers. Luciferase reporter assays confirmed that chondrocyte enhancers characterized in this study exhibited enhancer activity which may be modulated by DNA methylation and SOX9 overexpression. Altogether, these integrated data illustrate the cross‐talk between different epigenetic mechanisms during chondrocyte differentiation.

**Abbreviations**
ACAN: aggrecan
ChIP‐seq: chromatin immunoprecipitation
COL2A1: collagen type II alpha 1 chain
DMEM: Dulbecco's modified eagle medium
ECM: extracellular matrix
GO: gene ontology
hMSC: human mesenchymal stem cell
IGV: integrative genome viewer
lincRNA: long non coding RNA
MACS2: model‐based analysis of ChIP‐seq 2
miRNA: microRNA
OA: osteoarthritis
PBS: phosphate buffered saline
RUNX2: runt‐related transcription factor 2
SOX9: sex determining region Y box 9

## 1 INTRODUCTION

Chondrogenesis is the process of differentiation of mesenchymal progenitors into chondrocytes. Articular cartilage, present in synovial joints, comprises an extracellular matrix secreted by chondrocytes and has an important function in aiding the mobility of joints. As the only cell type present in articular cartilage, adult articular chondrocytes are responsible for the homeostasis of cartilage.

During embryogenesis, the skeletal system originates from the mesoderm germ layer. Mesenchymal progenitors differentiate into chondrocytes to form temporary cartilage. During endochondral ossification, these cells generally undergo apoptosis to be replaced by bone. However, cartilage at synovial joints does not ossify and remains throughout the life. Hypertrophic chondrocytes bound for ossification have high expression of *COL10A1* and osteoblast markers such as *RUNX2*, and low expression of cartilage‐specific genes such as *COL2A1* and *SOX9*.^1^, ^2^ Chondrogenesis is a multi‐step tightly regulated process mediated by growth and transcription factors, with the SOX9 transcription factor instrumental to the progression of chondrogenic differentiation 3 although not initiation.^4^ Gene expression during chondrogenesis is in part regulated by dynamic epigenetic mechanisms such as DNA methylation and histone modifications.^5^, ^6^ MicroRNAs (miRNAs) and long non‐coding RNAs (lncRNAs) also play a role in chondrogenesis.^7^, ^8^, ^9^ Genome‐wide histone modification changes have been observed during in vitro differentiation of MSCs into chondrocytes.^10^ As well as development, epigenetic mechanisms are also known to be involved in disease. *Cis*‐regulatory elements such as gene enhancers have been shown to be disrupted in cartilage pathologies. Deletions in a distal regulatory region of the *SOX9* transcription factor gene and within the *SOX9* gene itself both lead to campomelic dysplasia in humans.^11^, ^12^ Mutations in enhancers of collagen genes are also associated with chondrodysplasias.^13^, ^14^ Osteoarthritis (OA), an age‐related cartilage degenerative disease, has a strong genetic component and to date, the vast majority of polymorphisms that confer an increased risk are located in non‐coding regions of the genome, including enhancers.^15^, ^16^ There is evidence that the OA phenotype may be linked to the reactivation of developmental pathways.^17^ Articular cartilage affected by OA shows gene expression changes reminiscent of hypertrophic chondrocytes.^1^, ^18^ These studies demonstrate that epigenetic mechanisms regulate gene expression in numerous biological processes. However, how these mechanisms affect gene expression is not fully understood in cartilage development and disease.

Mesenchymal stem cells (MSCs) are able to differentiate into chondrocytes and have been used to study chondrogenesis in vitro. Tissue engineering solutions to cartilage repair include autologous chondrocyte implantation, cartilage autografts, and injection of MSCs into the damaged site.^19^, ^20^ However, these methods are not widely used and complications can arise from their application. Further knowledge of the regulatory processes that control gene expression during chondrocyte development is required to develop and improve models for cartilage regeneration. Usage of in vitro models for human chondrogenesis is crucial for understanding the changes that occur during the normal development of human cartilage. Additionally, as in vitro models are used extensively for the study of chondrogenesis, it is important to establish how similar models are to each other and to in vivo chondrogenesis.

In this study, histone ChIP‐seq (H3K4me3, H3K4me1, H3K27ac, H3K27me3, and H3K36me3) was performed in a scaffold‐free in vitro model of human MSC (hMSC) chondrogenesis.^21^ Analysis of histone ChIP‐seq data revealed that large scale chromatin state changes occur during chondrogenesis and chondrocytes acquire cell‐type‐specific enhancers upon differentiation. Integration of chromatin states with genome‐wide DNA methylation data demonstrated that de‐methylated CpG sites are located within H3K27ac and H3K4me1 marked enhancers during chondrogenesis. Motif analysis revealed that chondrocyte enhancers contain *SOX9*‐binding motifs. Altogether, our study provides a comprehensive analysis of the global epigenetic changes during MSC chondrogenesis and highlights the role of enhancers in defining cell‐type specificity.

## 2 MATERIALS AND METHODS

### 2.1 hMSC culture and chondrogenesis

Bone marrow aspirates (donor n = 2, female, ages 22 & 24) were purchased from LONZA and hMSCs were isolated by adherence to tissue culture flasks for 24 hours. hMSCs were phenotyped by flow cytometry^22^ and confirmed to have osteoblastogenic and adipogenetic potential as well as chondrogenic. Stem cells were cultured and differentiated into chondrocytes as previously described.^23^

### 2.2 Isolation of chondrocytes from cartilage‐like disc

Cartilage discs were digested at Day 14 of chondrogenesis, a time point at which chondrocytes have been determined to be fully differentiated in a pellet model of chondrogenesis.^24^ Cartilage discs were digested first with 1.5 mL of hyaluronidase (1 mg/mL in sterile PBS) for 15 minutes at 37°C then with 1.5 mL of trypsin (2.5 mg/mL in sterile PBS) at 37°C for 30 minutes. The discs were finally digested with collagenase (2 mg/mL in DMEM media) for 1‐1.5 hours at 37°C until fully digested and the matrix was no longer visible. The digested cartilage containing media was passed through a 100 μm cell strainer to remove any remaining matrix. Each cartilage disc yielded ~250,000‐500,000 cells and multiple discs were pooled together during extraction.

### 2.3 Chromatin extraction and ChIP‐seq

hMSCs were harvested from monolayer culture using trypsin. Chromatin from hMSCs and differentiated chondrocytes were extracted using the Diagenode iDeal histone ChIP‐seq kit (Diagenode SA, Ougrée, Belgium). Extracted chromatin was sonicated using a Diagenode Bioruptor Standard or Bioruptor Pico to an average size of 200‐500 bp, using 15 sonication cycles (30s on/30 seconds off). ChIP‐seq grade premium antibodies were purchased from Diagenode: H3K4me3 (included in the Diagenode iDeal histone ChIP‐seq kit), H3K4me1 (Cat. no. C15410194), H3K27ac (Cat. no. C15410196), H3K27me3 (Cat. no. C15410195), and H3K36me3 (Cat. no. C15410192). Chromatin immunoprecipitation was performed following the Diagenode iDeal histone ChIP‐seq protocol using chromatin from 1 million cells and 1μg antibody per ChIP. Immunoprecipitated DNA was purified using Agencourt AMPure XP beads (Beckman Coulter (UK) Ltd, High Wycombe, UK). For one hMSC chondrogenesis replicate ChIP‐seq, DNA sequencing libraries were generated using Diagenode MicroPlex v2 kit and single‐ended reads of 50 bp length were generated on an Illumina HiSeq 2500 (Illumina Inc, San Diego, USA). The second experimental replicate was prepared using the NEBNext Ultra II kit (New England Biolabs, Hitchin, UK) and sequenced using an Illumina NextSeq 500 platform, generating 75 bp single‐end reads. For both replicates, 30‐65 million reads were generated per sample (Table S1).

### 2.4 Luciferase reporter assays

Putative enhancer regions were amplified from human genomic DNA using the primers listed in Table S2 and cloned into the pCpGL‐EF1 plasmid. This plasmid has been modified from the CpG‐free pCpGL‐basic luciferase plasmid by the addition of the EF1 CpG‐free promoter upstream of the luciferase gene^25^ and can thus be used to analyze DNA methylation effects on non‐promoter regulatory regions. Plasmids were transformed into GT115 *E coli* (Invitrogen) and DNA isolated using the PureYield Plasmid Midiprep system (Promega). Plasmid DNA was in vitro methylated using CpG Methyltransferase (*M SssI*, New England Biolabs), with the efficiency of methylation assessed by digestion using *HpaII *and *HhaI* methylation‐sensitive restriction enzymes (NEB). The effect of SOX9 on enhancer activity was assessed by transfection with a SOX9 overexpression plasmid (pUT‐FLAG‐SOX9).^26^ A luciferase reporter (4COL) containing four copies of the Col2a1 48‐bp enhancer was used to confirm SOX9 overexpression.^27^ SW1353 chondrosarcoma cells were seeded at a cell density of 5 × 10^3^ per well in 96‐well plates. After 24 hours, cells were co‐transfected with 100 ng (DNA methylation) or 50 ng (*SOX9* overexpression) of the relevant pCpGL‐EF1 plasmid and 6ng (DNA methylation) or 1.5 ng (*SOX9* overexpression) of pRL‐TK Renilla control plasmid and 0.3 μL of FuGENE HD reagent (Promega) per well. Cells were lysed 24hrs post‐transfection and luciferase and the renilla activity measured using the Dual‐Luciferase Reporter Assay kit on a GloMax‐Multi reader. Three experiments with six replicates each were performed for each construct and *luciferase*/*renilla* activity normalized to for the empty the pCpGL‐EF1vector.

### 2.5 ChIP‐seq analysis and chromatin state learning

Quality control of sequencing reads was performed using FastQC (v.0.11.5). All reads passed quality thresholds. Reads were aligned to the reference human genome hg38 using Bowtie2 (v.2.2.4).^28^ MACS2 (v.2.1.0.2)^29^ was used to call broad peaks (parameters*–broad* and *–no‐model*) using input samples as controls. The ngs.plot program (v.2.61)^30^ was used to visualize peak enrichment across the genome and at gene expression levels. An Illumina whole‐genome expression array Human HT‐12 V4 was used to determine gene expression levels prior and post chondrogenesis.^23^ Normalized gene expression signals were categorized into low (signal < 7; 1st quarter), medium (signal between 7 and 9) or high expression (signal > 9; 3rd quarter; Table S3).

ChromHMM (v.1.12)^31^ was used to train a 16 state model on all histone marks assayed. The number of states was arrived at by running the model with different numbers of states until the separation of chromatin states was seen; as described by the Roadmap Epigenomics Project.^32^ The Integrative Genomics Viewer (IGV) was used to visualize chromatin state tracks.^33^ Global chromatin state changes between hMSC and differentiated chondrocytes were visualized using the riverplot package in R. Gene ontology (GO) terms for chromatin states were found using the GREAT tool with default settings.^34^

Mouse SOX9 ChIP‐seq data (GEO http://www.ncbi.nlm.nih.gov/geo/query/acc.cgi?acc=GSE69109) were aligned to mm10 using Bowtie2 (default settings). Aligned reads were converted to hg38 using the UCSC liftOver tool and narrow peaks were called using MACS2 (v.2.1.0.2) using input samples as control.

### 2.6 Chromatin state comparisons with roadmap epigenomics cell‐types

Chromatin state coordinates from our study were converted to hg19 using UCSC liftOver as Roadmap data were aligned to hg19. Similarity analysis between equivalent chromatin states across hMSC, chondrocyte, and Roadmap cell‐types was performed using the Jaccard index and hierarchical clustering. Roadmap chromatin state data are available to download from the project website (http://www.roadmapepigenomics.org/). For comparisons with human articular chondrocyte enhancers, histone ChIP‐seq data from human fetal and adult articular cartilage were accessed from http://www.ncbi.nlm.nih.gov/geo/query/acc.cgi?acc=GSE111850.^35^

### 2.7 Integration with DNA methylation

An Infinium HumanMethylation450 BeadChip array was used to quantify DNA methylation in the Transwell model of chondrogenesis,^36^ GEO dataset http://www.ncbi.nlm.nih.gov/geo/query/acc.cgi?acc=GSE129266. CpG probes from the 450K methylation array were based on human reference genome hg19; therefore, CpG coordinates from the array were first converted to hg38 and intersected with chromatin state coordinates from hMSC and differentiated chondrocytes. A Chi‐square test with 1000 Monte Carlo permutations was used to test the independence of de‐methylated CpG distribution in enhancers. All plots were generated using the ggplot2 package in R.

### 2.8 Motif analysis

The MEME suite of tools was used for de novo motif searching.^37^ The analysis of motif enrichment (AME) tool within MEME was used to assess the relative enrichment of SOX9‐binding motifs found in the footprintDB database^38^ in new chondrocyte enhancers compared to constant enhancers.

### 2.9 Data availability

ChIP‐seq data have been deposited http://www.ncbi.nlm.nih.gov/geo/query/acc.cgi?acc=GSE129031. The chondrogenesis 450k DNA methylation array data can be found in http://www.ncbi.nlm.nih.gov/geo/query/acc.cgi?acc=GSE129266. The chondrogenesis transcriptome analysis using Illumina whole‐genome expression array Human HT‐12 V4 is available upon reasonable request from the authors.

## 3 RESULTS

### 3.1 Chromatin state changes during chondrogenesis

Bone marrow‐derived hMSCs were differentiated into chondrocytes over 14 days using an in vitro Transwell model of chondrogenesis. This scaffold‐free model produces a cartilaginous disc which expresses matrix components such as type II collagen and sulphated glycosaminoglycans. The produced matrix assembles cartilage collagens and generates a robust collagen network with the prerequisite covalent cross‐links.^39^ Chondrogenic genes such as *SOX9* have been shown to be induced during the differentiation of hMSCs using this established and reproducible model of chondrogenesis.^8^, ^21^, ^23^ We observed the upregulation of markers of articular chondrocytes such as *COL2A1*, *TNBS4*, *PRG4*, but also markers of hypertrophic chondrocytes such as *COL10A1*, *PTH1R*, and *ALPL* (Figure S1).^40^, ^41^, ^42^

**Figure 1:**
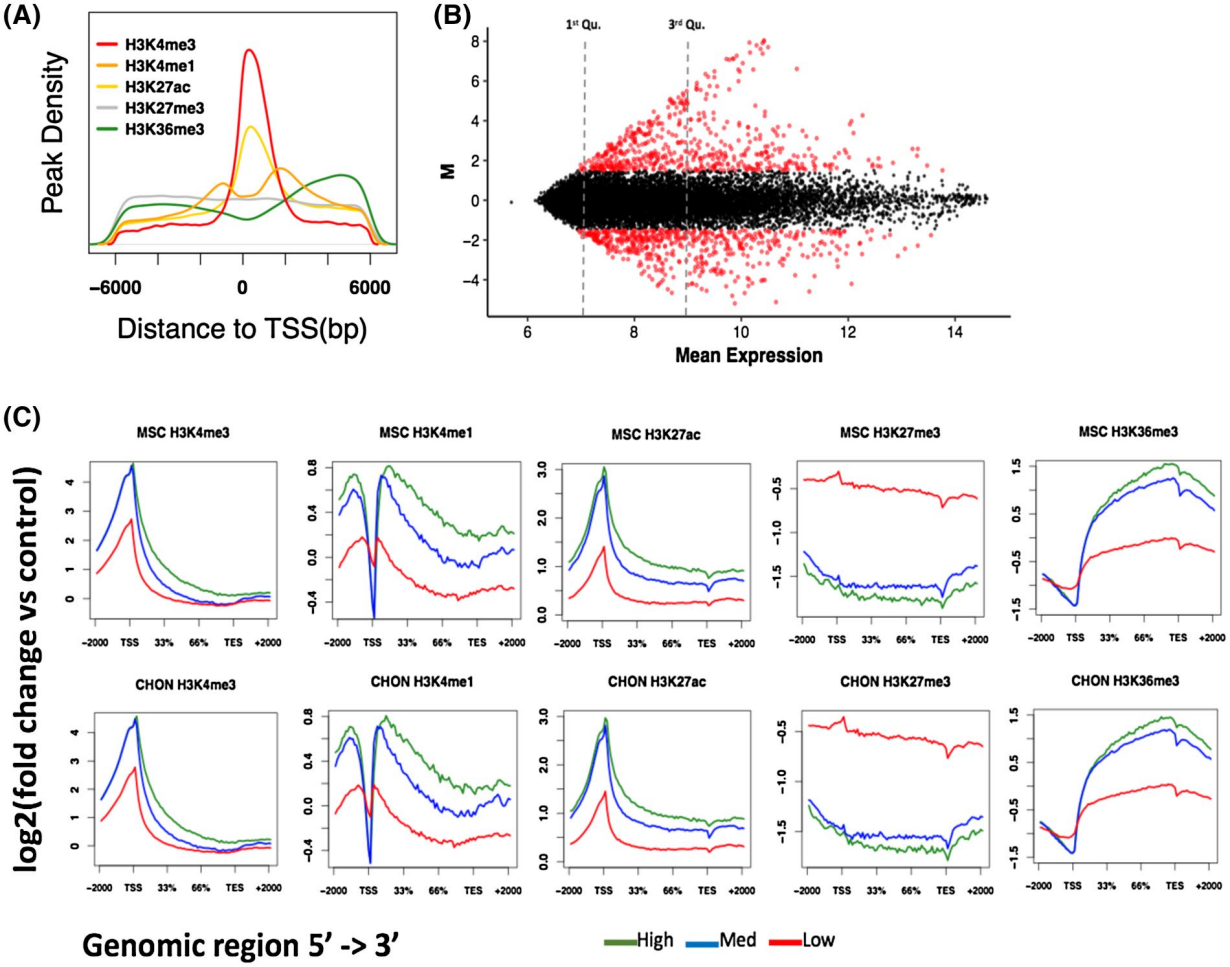
Correlation of histone mark enrichment with gene expression in hMSCs and differentiated chondrocytes. A, Density of histone mark peaks around the TSS (±6 kb). Peaks were called using MACS2 using input samples as controls for background noise. B, MA (log ratio—average expression) plot of differentially expressed genes between hMSCs and differentiated chondrocytes. Gene expression was measured using a cDNA microarray and gene expression is reported as normalized signals. Significantly differentially expressed genes are plotted in red. C, Histone mark enrichments in genes categorized as high expression (expression value > 9; upper quartile), medium expression (expression value > 7 and < 9) and low expression (expression value < 7; lower quartile). Histone marks were associated/overlapped to genes using the ngs.plot tool and plots were generated in R

Histone modifications H3K4me3, H3K4me1, H3K27ac, H3K27me3, and H3K36me3 were assayed in hMSCs and differentiated chondrocytes (Day 14) using ChIP‐seq. These histone marks were selected to reflect a wide range of regulatory states. H3K4me3 commonly marks active promoters, H3K4me1 and H3K27ac are found at active enhancers, H3K36me3 are located at actively transcribed regions and H3K27me3 marks transcriptionally repressed regions. The genome‐wide profiles of each histone mark were as expected; the density of each histone mark differs across the genome with the active promoter mark H3K4me3 showing a high density of peaks close to transcriptional start sites (TSS; Figure 1A). Histone modifications are known to influence gene transcription; therefore, histone mark enrichments were correlated with the expression levels of genes in hMSCs and differentiated chondrocytes (Figure 1B; Table S3). Gene expression in hMSCs and differentiated chondrocytes measured by microarray were stratified into groups of low, medium, and highly expressed genes (Table S3). Average read coverages of histone marks across each group were plotted and as expected histone marks and as expected, histone marks typically associated with transcriptional activity were enriched in highly expressed genes (Figure 1C). In contrast, the transcriptionally repressive mark H3K27me3 showed a greater enrichment in genes with low expression levels in differentiated chondrocytes. This demonstrates that the histone ChIP‐seq generated in hMSCs and differentiated chondrocyte exhibit expected genome‐wide profiles and gene expression associations.

**Figure 2:**
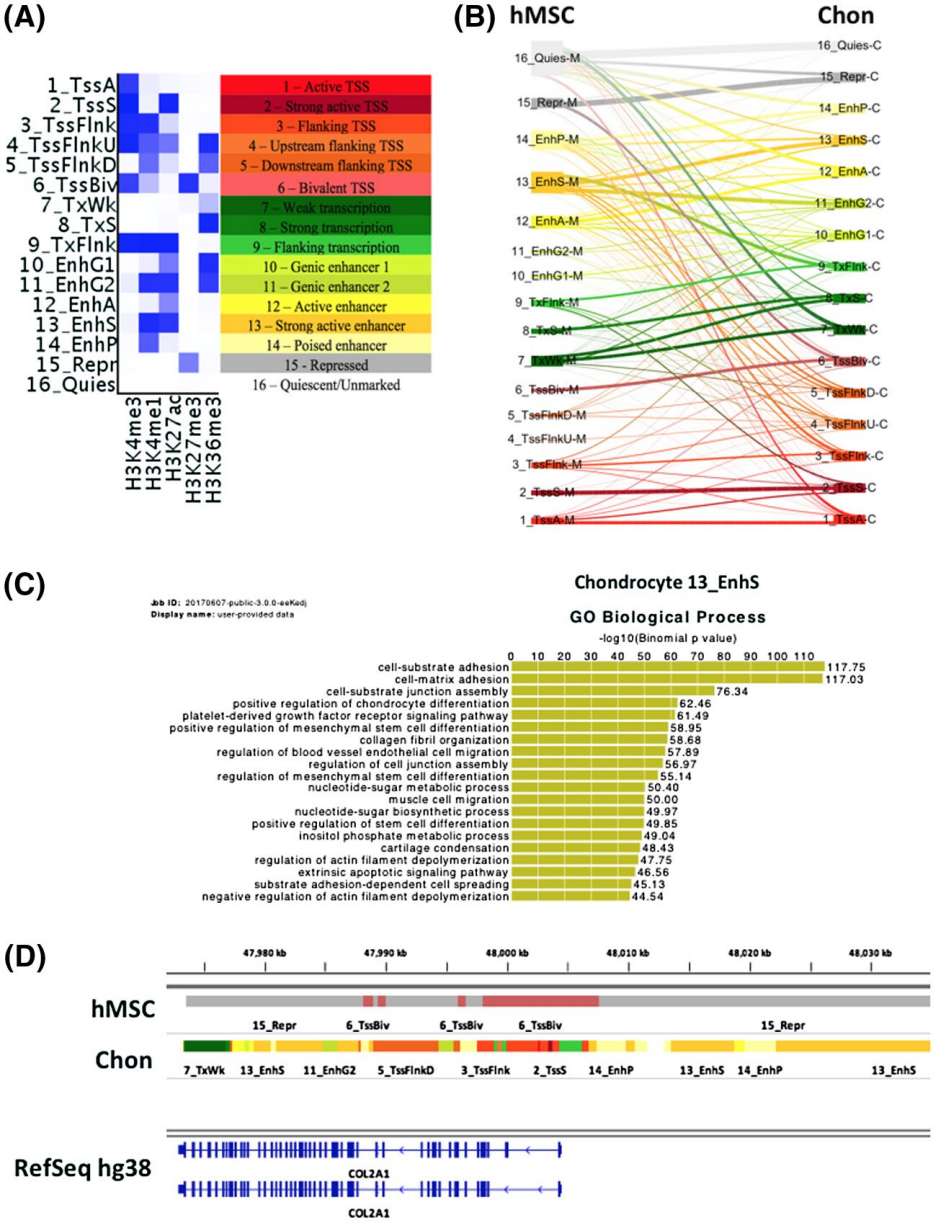
A 16 chromatin state model generated from hMSC and differentiated chondrocyte histone ChIP‐seq. A, Chromatin state model with annotated states. The chromatin state model was generated using the ChromHMM tool with all hMSC and differentiated chondrocyte data using input controls. Intensity of blue within the heatmap represents the model emission probabilities (Table S4). B, Genome‐wide changes in chromatin states during hMSC chondrogenesis. The thickness of the lines represents the frequency of chromatin state changes from hMSC to chondrocytes (Figure S2). C, GREAT biological process GO terms for the chondrocyte strong enhancer (13_EnhS) state. Genome coordinates of the 13_EnhS were used as input into the GREAT tool which associates cis‐regulatory elements with genes. D, IGV genome browser view of hMSC and chondrocyte chromatin states around the COL2A1 gene. Colors for the ChromHMM defined chromatin states are as in panel A

Combinations of histone modifications can reveal more information about the regulation of gene expression compared to singular histone marks.^43^ Regulatory elements and chromatin states may be defined by the co‐occurrences of specific histone marks.^44^ A 16 chromatin state model was trained on the hMSC and differentiated chondrocyte ChIP‐seq data using ChromHMM (Figure 2A). The model yielded a range of chromatin states known to be associated with the histone modifications assayed in this study (model emission probabilities are shown in Table S4). This included promoter states, actively transcribed states and enhancer elements.^32^ Large scale changes in chromatin states were observed between hMSCs and differentiated chondrocytes, particularly with regards to the quiescent and repressed states becoming transcriptionally active (Figures 2B, S2), demonstrating that genome‐wide histone modification changes occur in the epigenome during chondrogenesis. To elucidate how chromatin states affect gene expression, the GREAT tool^34^ was used to retrieve gene ontology (GO) terms for each chromatin state. GO terms associated with genes linked to each of the defined chromatin states were non‐specific to cell‐type and mostly encompassed general cell functions, the exception being enhancer states (Figure S3‐S17). In differentiated chondrocytes, the strong active enhancer state (characterized by high enrichment of H3K4me1 and H3K27ac; state 13_EnhS) yielded GO terms related to chondrogenesis and cartilage function (Figure 2C). Previous studies have demonstrated that gene enhancers are cell‐type specific and play an important role in regulating cell‐type specific processes.^45^ Accordingly, chondrocyte enhancers defined in this study are associated with chondrogenesis related terms, more than promoter or gene transcription chromatin states. Chromatin state changes can clearly be observed around genes that show gene expression changes. For example, we observed the histone modification around the *COL2A1* gene switching from repressed/inactive in hMSCs to transcriptionally permissive in chondrocytes (Figure 2D).

### 3.2 Comparison to roadmap epigenomics cell types

**Figure 3:**
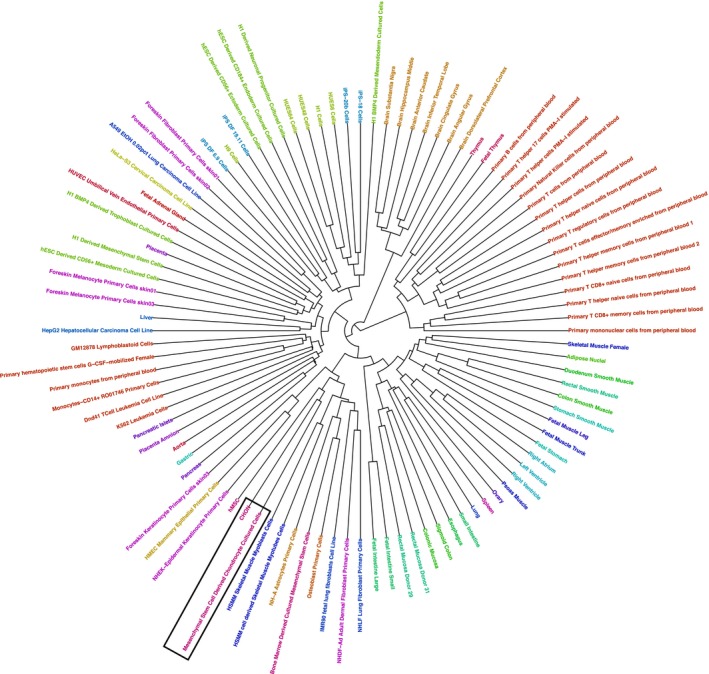
Clustered similarity heatmap of H3K4me1 and H3K27ac enhancers in hMSC and differentiated chondrocytes vs Roadmap cell types. The Jaccard similarity coefficient was calculated between state 13_EnhS in the Transwell chondrogenesis model and 9_EnhA1 in Roadmap 18 state model across cell types. Both states are characterized by high enrichment of H3K4me1 and H3K27ac. Stem cells and differentiated chondrocytes from this study are labeled “hMSC” and “CHON”, respectively. Chondrocytes are indicated by black boxes. A list of Roadmap cell types and their ID codes can be found on the Roadmap project website (http://www.roadmapepigenomics.org)

Several large‐scale consortia have aimed to characterize the epigenomes of various cell‐types including the NIH Roadmap Epigenomics project,^32^ which defined chromatin states in 127 cell‐types, 98 of which also included the active enhancer mark, H3K27ac. Roadmap cell‐types contained bone marrow‐derived hMSCs and differentiated chondrocytes; therefore, we sought to determine whether the epigenome of our chondrocytes was comparable to those included in the Roadmap project. We compared our 16 chromatin states to the equivalent states of the 18 state model generated by the Roadmap project for their 98 cell‐types that contained the H3K27ac active enhancer mark (Figure S18).The Jaccard similarity coefficient was used to compare equivalent chromatin states across all cell‐types in a pairwise manner. When individual chromatin states except for enhancers were investigated there appeared to be no apparent clustering of cells by type or origin (Figure S19). In contrast, when H3K27ac and H3K4me1 marked enhancers (labeled 13_EnhS in transwell chondrogenesis chromatin state model and 9_EnhA1 in Roadmap 18 state model) were explored, cells clustered with other more closely related cell‐types (Figure 3). Our differentiated chondrocytes (“CHON” in Figure 3) clustered together with the BM‐MSC differentiated chondrocytes from the Roadmap project,^10^ demonstrating a higher level of similarity to each other than to all other cell types. The Roadmap bone marrow‐derived hMSCs and hMSCs in this study were closely related, contained within a small cluster of primary culture cells consisting of chondrocytes, myocytes, osteoblasts, and fibroblasts (Figure 3). These data corroborate previous studies that report that enhancers are distinct between cell‐types, more than any other regulatory features such as gene promoters.^45^, ^46^ Further, enhancers in chondrocytes from different sources showed higher similarity compared to other cell‐type enhancers. Thus, there is a chondrocyte‐specific epigenome based on gene enhancers that can be detected despite differences in chondrogenesis models, laboratory, and MSCs donors.

### 3.3 Comparison to chondrocyte epigenomes

**Figure 4:**
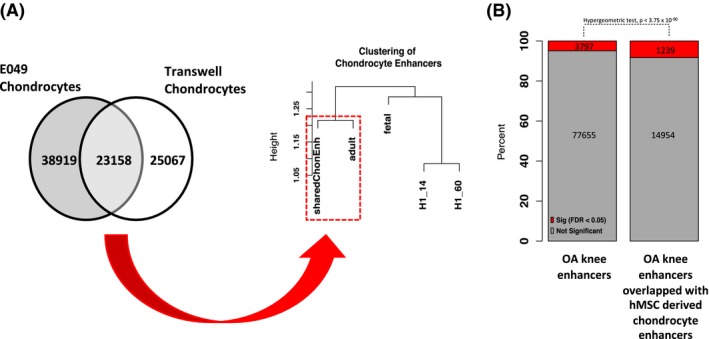
Comparison of shared hMSC‐derived chondrocyte enhancers to human articular chondrocyte and H1‐derived chondrocyte enhancers. A, There were 23 158 shared enhancer regions between differentiated chondrocytes defined in this study and Roadmap E049 enhancers. This shared hMSC‐derived chondrocyte enhancer signature was more similar to adult articular chondrocytes (dashed red box) compared to fetal articular chondrocytes or H1‐derived chondrocytes, as determined by the Jaccard similarity index. Jaccard similarity values were converted to Euclidean distance for hierarchical clustering. The height of the dendrogram represents the distance between samples; nodes joining at lower heights are more similar compared to those joining at greater heights. B, Overlap of shared hMSC‐derived chondrocyte enhancers with enhancers defined in OA knee cartilage. The proportion of enhancers dysregulated in knee OA is shaded in red. A hypergeometric test shows there was a significant overrepresentation of dysregulated enhancers that overlapped with shared hMSC‐derived chondrocyte enhancers (*P* < 3.75 × 10^−90^)

Roadmap chondrocyte enhancers were intersected with the chondrocyte enhancers identified in this study, resulting in a total of 23 158 enhancer regions common to both types of MSC‐derived chondrocytes (Table S5). We next compared these shared in vitro chondrocyte enhancers with enhancers identified in human fetal and adult articular cartilage^35^ using the Jaccard similarity coefficient to assess the concordance. The hMSC‐derived chondrocyte enhancer signature was more similar to adult articular chondrocytes compared to either fetal articular chondrocytes or H1 embryonic stem cell‐derived chondrocytes (Figure 4A). A study of differentially accessible chromatin regions in matched intact cartilage (outer region of the lateral tibial plateau) and damaged cartilage (inner region of medial tibial plateau) in OA knee found that enhancers were enriched in significantly differentially accessible regions.^47^ Of the 77 655 enhancers defined in the study by Liu et al, 14 954 overlapped with enhancers found in the shared enhancers in MSC‐derived chondrocytes. Furthermore, of the 3797 significantly differentially accessible enhancers between intact and damaged cartilage,^47^ 1239 were also found in differentiated chondrocyte enhancers (Figure 4B). This represents a significant overlap of differentially accessible enhancers in knee OA with enhancers in MSC‐derived chondrocytes (hypergeometric test, *P* < 3.75 × 10^−90^). This confirms the finding by Lui et al, that dysregulated enhancers in OA are enriched in cell‐type‐specific enhancers.

### 3.4 DNA methylation at gene enhancers

**Figure 5:**
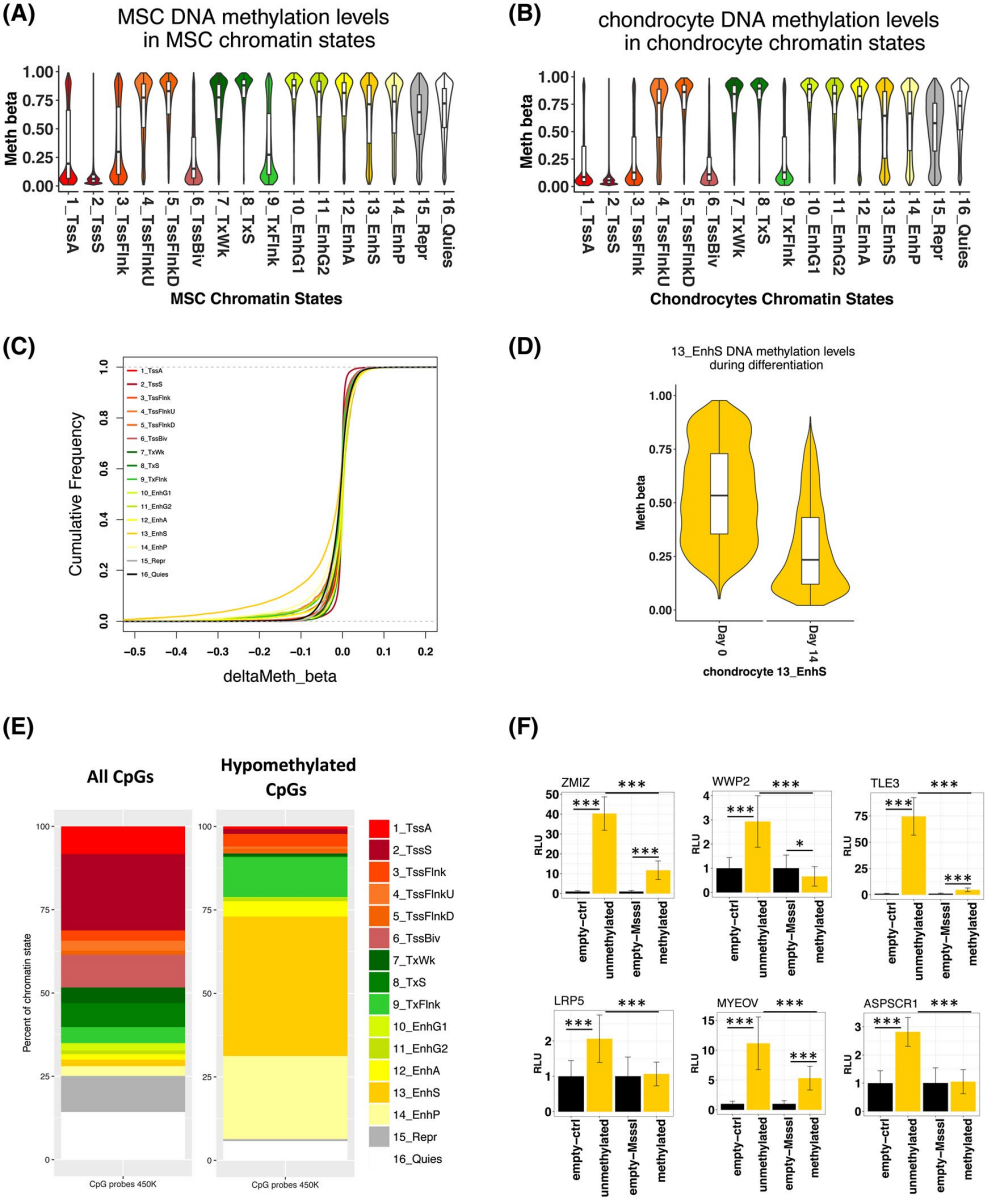
DNA methylation in chondrocyte chromatin states. A,B, Methylation levels of CpGs in the hMSC and chondrocyte chromatin states, respectively. CpG genome coordinates were intersected with chromatin states using BEDTools intersect. C, Empirical cumulative frequency plot of methylation changes (beta values) in chondrocyte chromatin states during hMSC chondrogenesis. D, Significantly methylated CpGs (FDR < 0.05) in between hMSCs and chondrocytes in chondrocyte chromatin states. E, The percentage of all CpGs on the 450k array in each chondrocyte chromatin state and the percentage of de‐methylated CpGs during chondrogenesis in chondrocyte chromatin states. F, Luciferase reporter assay with enhancer regions with and without DNA methylation (n = 6). Enhancers are labeled with their nearest gene. Significance levels: (*) *P*‐value < .05, (**) *P*‐value < .01, and (***) *P*‐value < .001. Error bars are ± standard deviation

Histone modifications are influenced by DNA methylation and vice versa during development.^48^ DNA methylation occurs at CpG sites in the genome and is typically associated with transcriptional repression. An Illumina Infinium HumanMethylation450K BeadChip array was used to measure DNA methylation. DNA methylation changes during the in vitro Transwell model of chondrogenesis were largely de‐methylation events that were associated with chondrogenesis‐related GO terms.^36^ We integrated the DNA methylation and ChIP‐seq data in order to investigate the DNA methylation changes in chromatin states during MSC chondrogenesis, focusing on the hypomethylated CpGs (94% of the significantly differentially methylated loci during chondrogenesis) since this is linked to gene transcription activation. Global methylation patterns reflect known trends (Figure 5A,B), for example, gene promoters tend to have low percentage methylation relative to the rest of the genome.^49^ We observed that enhancers marked by H3K4me1 and H3K27ac (13_EnhS state) were enriched for de‐methylated CpG sites (Figure 5C,D). Fewer than 2% of total CpGs probes present on the Infinium HumanMethylation450K BeadChip were located within chondrocyte chromatin state 13_EnhS (strong enhancers) yet remarkably 41.8% of de‐methylated CpGs were found in this chromatin state (Figure 5E), a highly significant over‐representation (Chi‐square test *P* < .001). We evaluated the effect of DNA methylation in six selected regions (Table S2) that acquired enhancer status during chondrogenesis in both our model and the chondrogenesis model in Roadmap and also overlapped with a H3K27ac signature during development in of human embryonic limbs.^50^ The nearest gene to each of these six regions (*ASPSCR1*, *TLE3*, *WWP2*, *ZMIZ1*, *LRP5*, and *MYEOV*) has also been reported to be important in chondrogenesis or cartilage‐related diseases. CpG sites around the *ASPSCR1* gene are differentially methylated in human knee OA compared to control.^51^ *TLE3* is a target of the SOX5, SOX6, and SOX9 trio of transcription factors important in chondrogenesis^52^ and is also involved in osteoblastogenesis.^53^ Wwp2, an E3 ubiquitin ligase, maintains cartilage homeostasis through the regulation of Adamts‐5.^54^ The gene is also the host for miR140, which is located toward the 3′ end of the *WWP2* gene and is co‐expressed with an isoform of *WWP2*. *MiR140* expression is unique to cartilage and plays an essential role in chondrocyte proliferation.^23^, ^54^, ^55^, ^56^ *ZMIZ1* is differentially expressed in OA chondrocytes following exposure to hyperosmotic conditions.^57^ *LRP5* is involved in MSC differentiation and cartilage degradation.^58^, ^59^ *MYEOV* is associated with multiple myeloma and may also be involved in abnormal bone homeostasis.^60^ Furthermore, CpG sites within these enhancers show a decrease of DNA methylation during chondrogenesis (Figure S20). The enhancer regions were cloned into a luciferase reporter vector with and without treatment of a CpG methyltransferase. Unmethylated enhancer regions showed increased enhancer activity compared to the empty vector control, confirming that regions classed as enhancers in our model exhibit enhancer activity. With the addition of a CpG methyltransferase, all regions showed a significant decrease in enhancer activity compared to unmethylated regions (Figure 5F).

### 3.5 Transcription factor binding at chondrocyte enhancers

**Figure 6:**
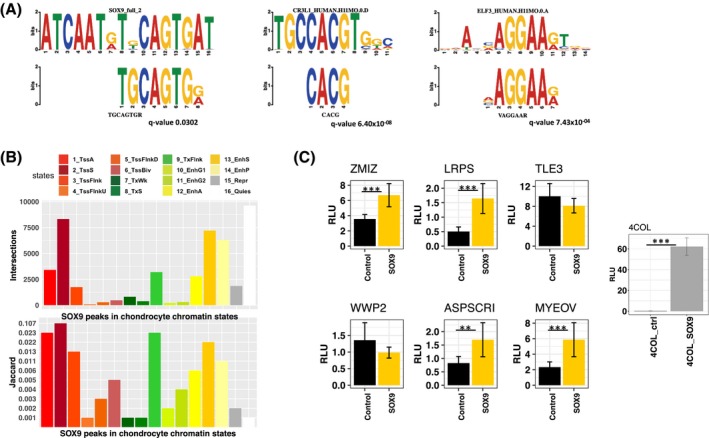
Transcription factor binding in chondrocyte chromatin states. A, Transcription factor motifs found in the chondrocyte strong enhancer state. TF motifs found include SOX9, CREB3L1 and ELF3 (Table S6). De novo motif analysis was performed using MEME. B, Numbers of SOX9 peaks derived from mouse rib chondrocyte ChIP‐seq data in chondrocyte chromatin states. Jaccard index of similarity between SOX9 peaks and chondrocyte chromatin states. C, Luciferase reporter assay with enhancer regions ± SOX9 overexpression (n = 6). Enhancers are labeled with their nearest gene. 4COL is a luciferase reporter containing four copies of the Col2a1 48‐bp enhancer and was used to confirm SOX9 overexpression^27^ Significance levels: (**) *P*‐value < .01 and (***) *P*‐value < .001. Error bars are ± standard deviation

Transcription factor binding occurs at gene enhancers to regulate gene expression.^61^, ^62^ Therefore, we determined whether chondrocyte enhancers defined in this study contained any transcription factor‐binding motifs. De novo motif searching of the chondrocyte strong enhancer state (13_EnhS) revealed SOX9‐binding motifs (Figure 6A). Motifs found in the strong enhancer state were highly specific to skeletal development with associations to skeletal diseases. For example, there was a positive match to a CREB3L1/OASIS motif, a transcription factor involved in the bone formation.^63^ Mutations in CREB3L1 have been linked to osteogenesis imperfecta.^64^, ^65^, ^66^ There was a match to ELF3, a transcription factor important during chondrogenesis^67^ and in cartilage degradation in OA.^68^, ^69^ Other matches include the HES and HEY family of transcription factors that are involved in chondrocyte hypertrophy during development 70, 71 (Table S6). The strong promoter state (2_TssS) also contained motifs belonging to transcription factors important in chondrogenesis such as SOX9 and ELF3 but also matched motifs of general transcription factors such as SP1 and the ETS family of transcription factors (Table S7).

SOX9 is a pivotal transcription factor driving chondrogenesis and interacts with promoters and enhancers to promote chondrogenesis.^72^ To further characterize chondrocyte enhancers, they were classified into two groups: new enhancers, defined by a change in chromatin state from quiescent or repressed to active enhancers during chondrogenesis, and constant enhancers; regions which were active enhancers both prior and post chondrogenesis. The analysis of motif enrichment (AME) algorithm implemented in the MEME suite of motif searching tools was used to contrast relative SOX9 motif enrichment in these two classes of enhancers found in chondrocytes. We found that both SOX9 motifs were significantly more enriched in the new enhancer class compared to the constant enhancer class (Figure S21). This suggests that enhancers have different properties depending on whether they acquired enhancer status upon differentiation or if they were enhancers beforehand.

To investigate whether SOX9 binds to motifs found in chondrocyte enhancers we used a publicly available mouse rib chondrocyte SOX9 ChIP‐seq dataset^72^ and converted the data to human genome coordinates. SOX9 is an evolutionary conserved transcription factor with conserved binding sites.^73^, ^74^, ^75^ De novo motif searching using lifted over SOX9 peaks recovered human SOX9 motifs (Table S7), as was the case with the original mouse analysis.^72^ This is evidence that the SOX9 binding site is conserved and the lifted over sequences contain SOX9 motifs, rather than being lifted over due to regional homology of the sequences around the motif. The majority of SOX9 peaks derived from mouse ChIP‐seq data were found in the chondrocyte strong promoter (2_TssS) state, strong active enhancer state (13_EnhS) state and quiescent state (16_Quies); the latter simply being due to the high percentage of the genome marked quiescent. Accounting for the size of chromatin states, there was more SOX9 enrichment in promoter and enhancer states (Figure 6B). This confirms that the chondrocyte promoters and enhancers identified in our study contain real and conserved SOX9‐binding sites. The impact of SOX9 overexpression was assessed on the previously cloned enhancer regions and a SOX9‐responsive Col2a1 enhancer reporter^27^ (Figure 6F). Four out of six enhancers exhibited increased enhancer activity with SOX9 overexpression (Figure 6C). All regions except one (nearest gene *TLE3*) have a SOX9‐binding site in the lifted over SOX9 ChIP‐seq data; as predicted, the *TLE3* region did not show increased enhancer activity upon SOX9 overexpression.

Previously, analysis of mouse Sox9 ChIP‐seq found that AP‐1 factors, Jun, and Fos were found to co‐localize with Sox9.^72^, ^76^ The authors found that whilst Sox9 and AP‐1 factors can form protein‐protein complexes, co‐localization primarily occurred through the binding of factors to the same binding sites. Positive matches to JUN and FOS motifs were also found in the de novo motif search of lifted over SOX9 peaks (Table S8), demonstrating that this mechanism is conserved between the two species.

## 4 DISCUSSION

There are numerous in vitro models of chondrogenesis and although some models utilize scaffolds for cells to grow, scaffold‐free models are reported to better reflect the conditions during in vivo chondrogenesis during development.^77^ Chondrocytes from this study were derived from a scaffold‐free chondrogenesis model using bone marrow‐derived hMSCs. Other scaffold‐free models include the micromass and pellet culture system. In contrast, chondrocytes from the Roadmap project were derived from human BM‐MSCs in a 3D alginate chondrogenesis model.^10^ Whilst there has been some gene expression comparisons between models,^78^, ^79^ no comparison has been made about changes in their epigenetic landscape. Here, we show that chondrocyte gene enhancers across two different models are highly concordant relative to other cell‐types. This is indicative of a unique chondrocyte epigenetic signature, independent of model and laboratory‐specific effects. Although hMSC‐derived chondrocyte enhancer concordance is evidence that chondrogenic models are reliable and comparable, further work is required to establish their likeness to in vivo chondrocytes. We observed both articular and growth plate chondrocyte gene expression markers in our differentiated chondrocytes. However, although the classical gene for hypertrophy, COL10A1, is upregulated, protein production or matrix deposition appears to be limited.^21^, ^39^ An upregulation of markers of hypertrophy is also commonly observed in pellet models of hMSC chondrogenesis.^80^, ^81^, ^82^ More work is needed to determine whether in vitro systems reflect chondrocytes which undergo endochondral ossification or articular chondrocytes found in adult synovial joints. However, we have identified in vitro chondrocyte enhancers that overlap with enhancers found in knee cartilage and corroborated that enhancers dysregulated in OA are more likely to be cell‐type‐specific enhancers.^47^ The concordance between enhancers identified between hMSC chondrogenesis models and OA suggests that in vitro models have an important role in studies into cartilage development and disease.

Combinations of histone modifications can define regulatory elements and regulate genes through modulating chromatin remodeling to allow or block access to transcription factors. However, histone modifications also rely on other epigenetic mechanisms such as DNA methylation and vice versa.^48^ Crosstalk between the two epigenetic mechanisms allows for greater control of gene transcription and it is important to consider histone modifications in the wider context of the whole epigenome. Traditionally, studies into DNA methylation focused on gene promoters where CpG islands are more likely to be found and array probe design is biased toward promoters. Although our data are extensive, we only compared ~ 450,000 (1.6%) of the ~28 million CpG sites in the human genome.^83^ Reduced representation bisulfite sequencing (RRBS) in chondrogenesis only identified limited CpG methylation changes in gene promoters.^10^ However, RRBS is heavily biased toward promoters and whole‐genome bisulfite sequencing remains the only method that can universally capture almost the entire DNA methylome. We show in this study that significant changes occur at distal gene enhancers during chondrogenesis. DNA demethylation at enhancer regions has also been observed during other stem cell differentiation processes, including differentiation of intestinal epithelium progenitors,^84^ hematopoietic stem cells 85 and embryonic stem cells 86 but also due to MSC age and culture conditions.^87^ DNA demethylation at enhancers is associated with the development of most human organs.^88^ Aberrant DNA methylation in enhancers has been implicated in diseases such as cancer^89^, ^90^, ^91^ and osteoarthritis (OA).^51^, ^92^

Motif discovery at chondrocyte enhancers recovered motifs of transcription factors known to be involved in cartilage development and diseases such as CREB3L1, ELF3, and SOX9. We utilized a mouse Sox9 ChIP‐seq dataset to assess whether enhancers defined in our study contained SOX9‐binding sites. SOX9 has a highly conserved DNA‐binding motif and function.^93^, ^94^, ^95^, ^96^ Therefore, we considered the liftover of mouse reads to human genome coordinates to be appropriate for our analysis. Indeed, we recovered a human SOX9 motif from lifted over peaks, illustrating that the DNA‐binding sites and motif of SOX9 are highly conserved between human and mouse. Using liftover, species‐specific SOX9‐binding information is lost but conserved sites are retained, these sites arguably being the most important, as evolutionary conservation is a marker of essentiality. We found SOX9 motifs in our chondrocyte enhancers via de novo motif searching as well as conserved SOX9 binding using mouse SOX9 ChIP‐seq. SOX9 acts in conjunction with transcription factors SOX5 and SOX6 in chondrogenesis,^97^ to bind to super enhancers promoting chondrogenesis.^98^ Super enhancers are loosely defined as multiple enhancers in close proximity exhibiting high levels of active enhancer markers such as H3K27ac or transcription factors (Pott and Lieb, 2015). SOX9 bound enhancers have previously been proposed to be important for defining the chondrocyte phenotype. Furthermore, mutations of Sox9‐binding motifs within distal *Acan* enhancers in transgenic mice resulted in a loss of chondrocyte‐specific expression.^99^

Enhancers are thought to regulate their target genes by forming a loop to physically contact the gene promoter within topologically associating domains^100^, ^101^; an interaction mediated by transcription factors.^102^ Gene enhancers can be located distal from their target promoters and therefore, target gene prediction can be challenging without chromatin conformation data.^103^ Although we have validated that enhancers identified in this study do, indeed, possess enhancer activity that may be modulated by DNA methylation and SOX9 binding, further functional work is required to elucidate their gene target(s) and importance in cartilage development. In this study, we show that enhancers are dynamic during chondrogenesis and may serve as potential targets for modulating hMSC differentiation.

To conclude, the integration of ChIP‐seq with methylation data revealed that gene enhancers are de‐methylated during an in vitro Transwell model of chondrogenesis. Comparison of chromatin states across hMSCs and chondrocytes generated in this study along with those from the Roadmap Epigenomics project revealed that enhancers marked by H3K4me1 and H3K27ac are more cell‐type specific compared to other chromatin states. Chondrocytes from the Epigenomics Roadmap project and this study showed a more similarity of enhancers with each other than other cell‐types despite being from different models. We have established that chondrocyte enhancers contain motifs to which SOX9 binds in vivo. Additional investigations are needed to elucidate further the epigenetic landscape of chondrocytes originating from other in vitro models and to determine whether these are comparable to the epigenome of human articular chondrocytes. A link between reactivation of developmental pathways and OA has been suggested^17^; more research is needed to fully explore the association between development and disease.

## 5 CONCLUSION

Human mesenchymal stem cells are able to differentiate into chondrocytes, the cell type found in cartilage, making them an accessible system to study gene regulation during this process. Epigenetic mechanisms such as histone modifications and DNA methylation together with transcription factor binding play a role in activating and repressing gene expression. In this study, we investigated the genome‐wide histone modification changes during chondrocyte differentiation. Integration of this data with DNA methylation and SOX9 transcription factor ChIP‐seq revealed epigenetic changes at gene enhancer elements. Regions of the genome that transition from non‐enhancers to enhancers in chondrocytes are enriched for SOX9 transcription factor‐binding sites. Luciferase reporter assays revealed that enhancer activity may be modulated by manipulating DNA methylation and SOX9 expression. This study has defined important regulatory elements in chondrocytes which could serve as targets for future mechanistic studies.

## CONFLICT OF INTEREST

None.

## AUTHOR CONTRIBUTIONS

Kathleen Cheung: Collection and/or assembly of data, data analysis and interpretation, manuscript writing. Matthew J. Barter: Collection and/or assembly of data, provision of study material or patients. Julia Falk: Collection and/or assembly of data. Carole Proctor: Conception and design. Louise N. Reynard: Conception and design, provision of study material or patients. David. A. Young: Conception and design, final approval of the manuscript.

## Data Availability

ChIP‐seq data have been submitted into the NCBI GEO data repository with accession http://www.ncbi.nlm.nih.gov/geo/query/acc.cgi?acc=GSE129031.
