# Supplementary material for: Histone ChIP‐Seq identifies differential enhancer usage during chondrogenesis as critical for defining cell‐type specificity
Source: FASEB J. 2020 Feb 14;34(4):5317–31. doi: 10.1096/fj.201902061RR (PMC7187454; doi:10.1096/fj.201902061RR)
Supplement: Supplementary file 1 [file FSB2-34-5317-s001.pdf]

Supplementary Figures

Fig. S1- Expression of chondrogenic markers from day 0 to day 14 of chondrogenesis.
Fig S2 – Frequency of chromatin state changes in hMSCs to chondrocytes.
Fig S3 - S17 - GO terms associated with genes linked to chondrocyte chromatin states
Fig. S18 - Equivalent chromatin states between Roadmap 18 state model and our 16 state model
Fig. S19 – Heatmaps of Jaccard similarity of cell types between equivalent chromatin states generated in this study and the Roadmap Epigenomics project.
Figure S20 – Methylation in the six enhancer regions selected for luciferase reporter assays.
Fig. S21 – Relative SOX9 enrichment in new enhancers compared to constant enhancers

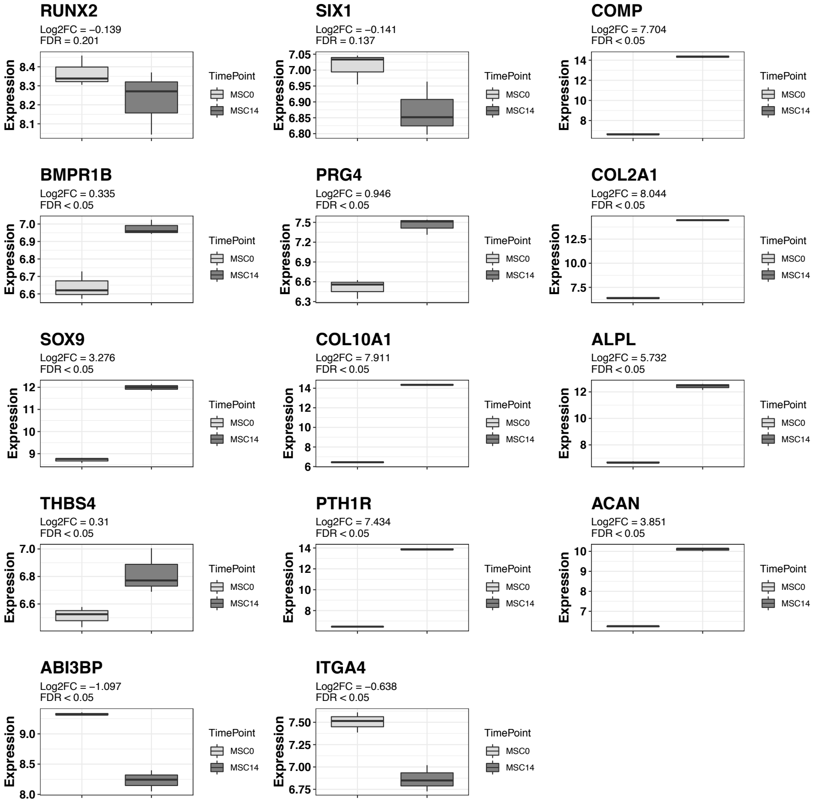

Supplementary Fig. S1 – Expression of chondrogenic markers from day 0 to day 14 of chondrogenesis. General chondrocyte markers: COMP, ACAN. Articular chondrocyte markers: COL2A1, SOX9, SIX1, PRG4, BMPR1B, ABI3BP. Hypertrophic/growth plate chondrocyte markers: ITGA4, ALPL, COL10A1, RUNX2, PTH1R

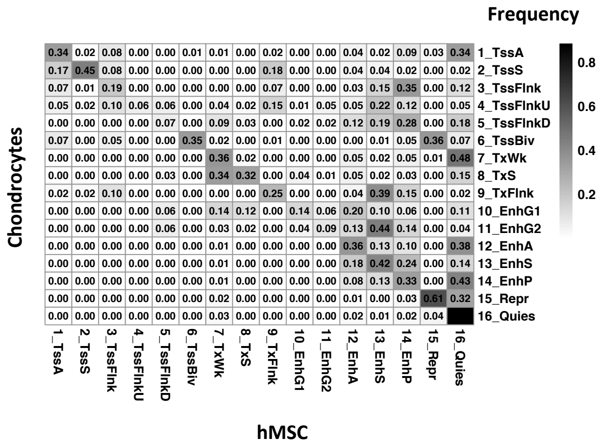

Supplementary Fig. S2 – Frequency of chromatin state changes in hMSCs to chondrocytes. The y axis shows the chondrocyte chromatin state and the x axis shows what state it was prior to chondrogenesis.

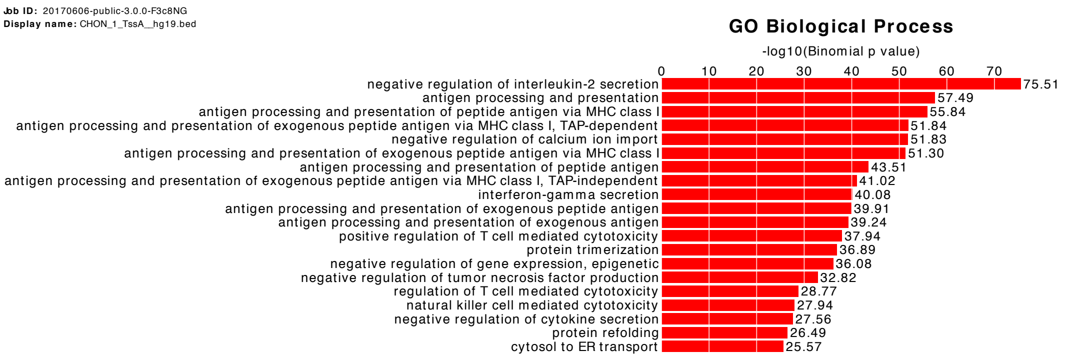

Figure S3 - GREAT GO terms for chondrocyte chromatin state 1_TssA

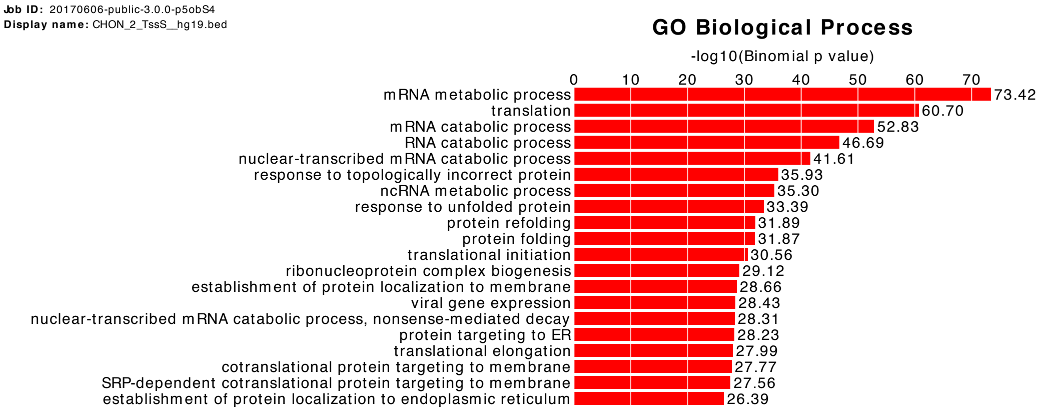

Figure S4 - GREAT GO terms for chondrocyte chromatin state 2_TssS

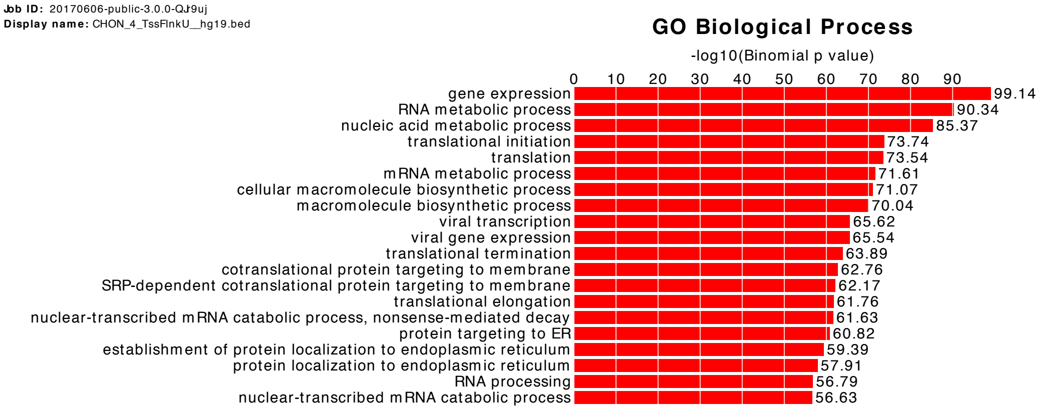

Figure S5 - GREAT GO terms for chondrocyte chromatin state 3_TssFlnk

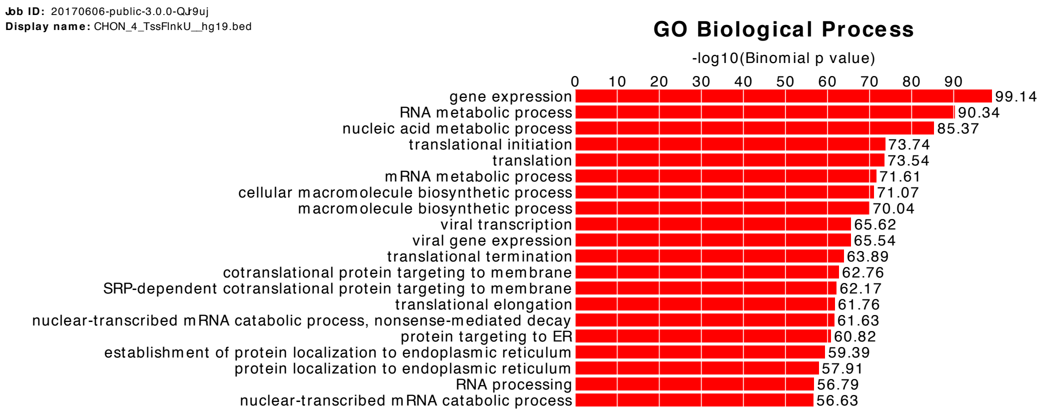

Figure S6 - GREAT GO terms for chondrocyte chromatin state 4_TssFlnkU

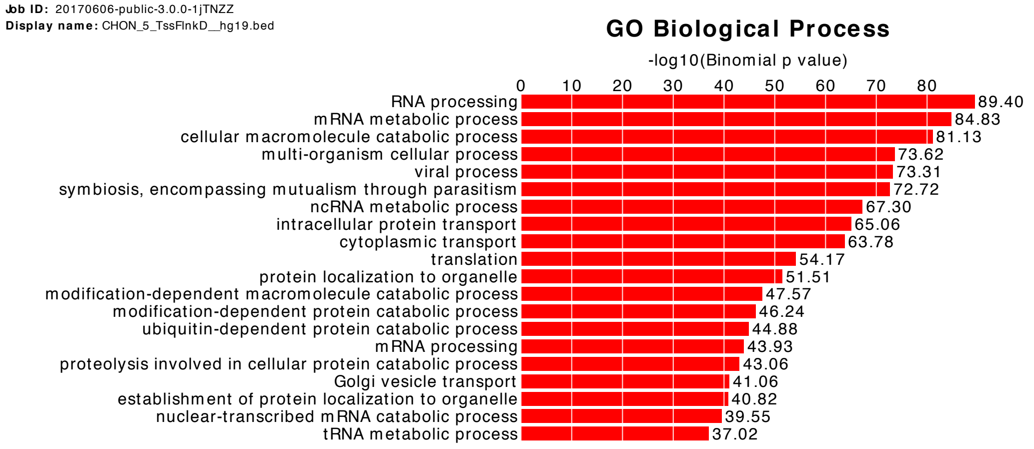

Figure S7 - GREAT GO terms for chondrocyte chromatin state 5_TssFlnkD

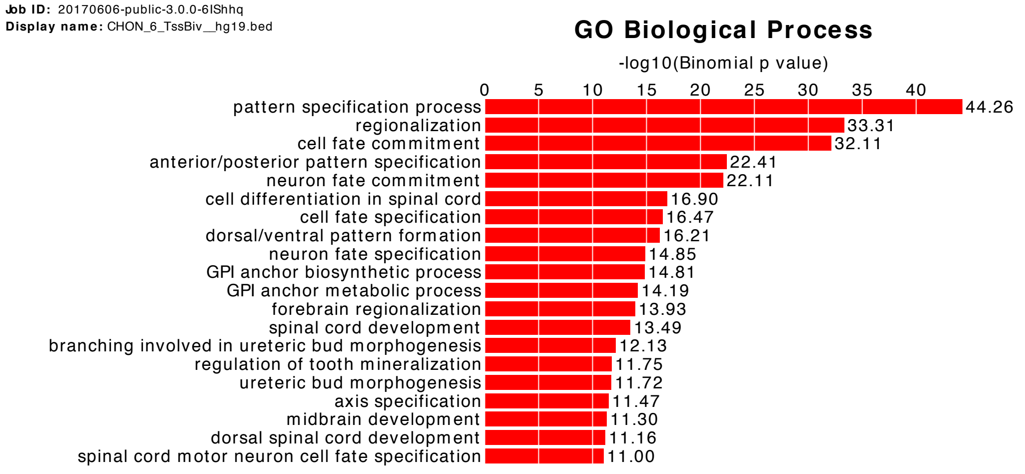

Figure S8 - GREAT GO terms for chondrocyte chromatin state 6_TssBiv

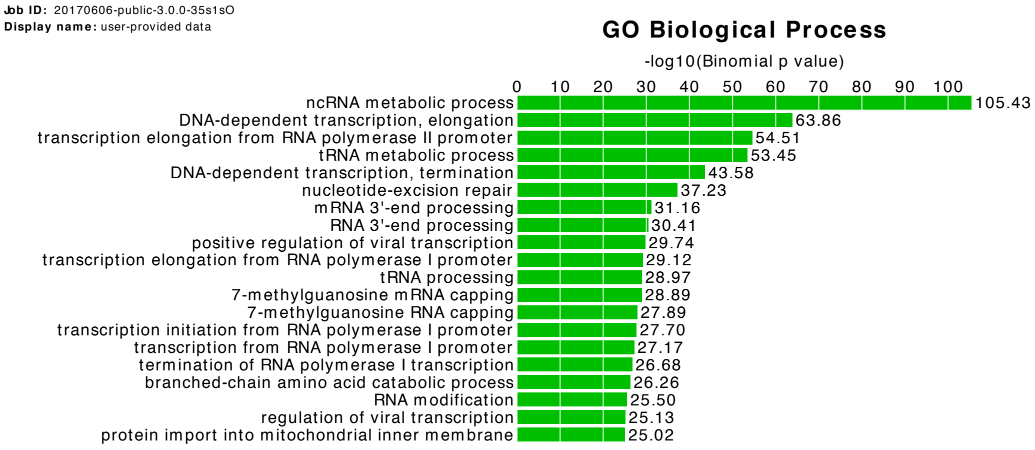

Figure S9 - GREAT GO terms for chondrocyte chromatin state 7_TxWk

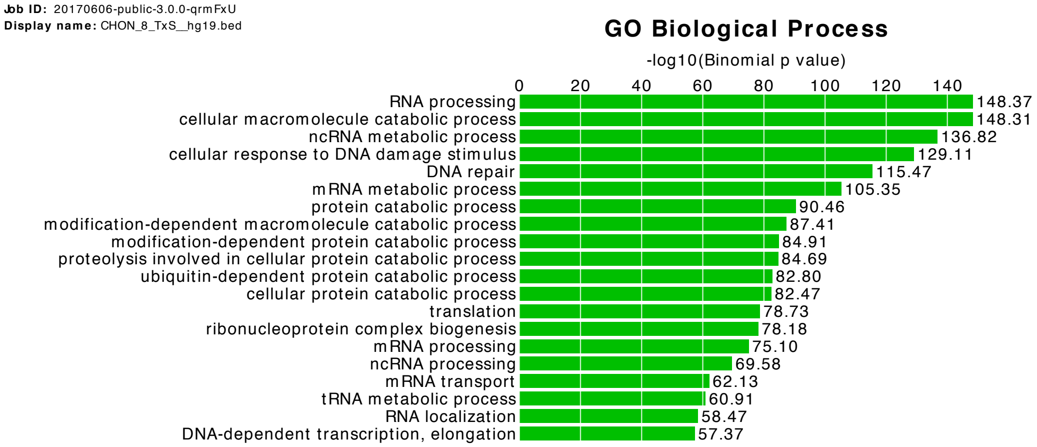

Figure S10 - GREAT GO terms for chondrocyte chromatin state 8_TxS

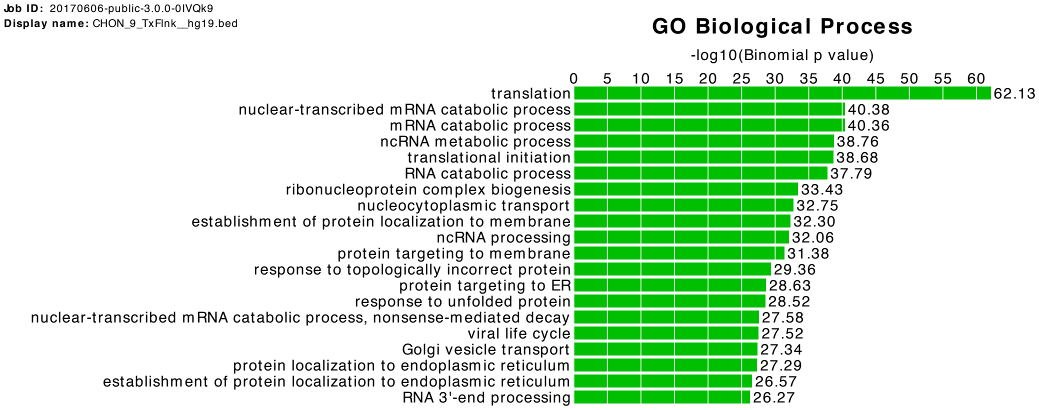

Figure S11 - GREAT GO terms for chondrocyte chromatin state 9_TxFlnk

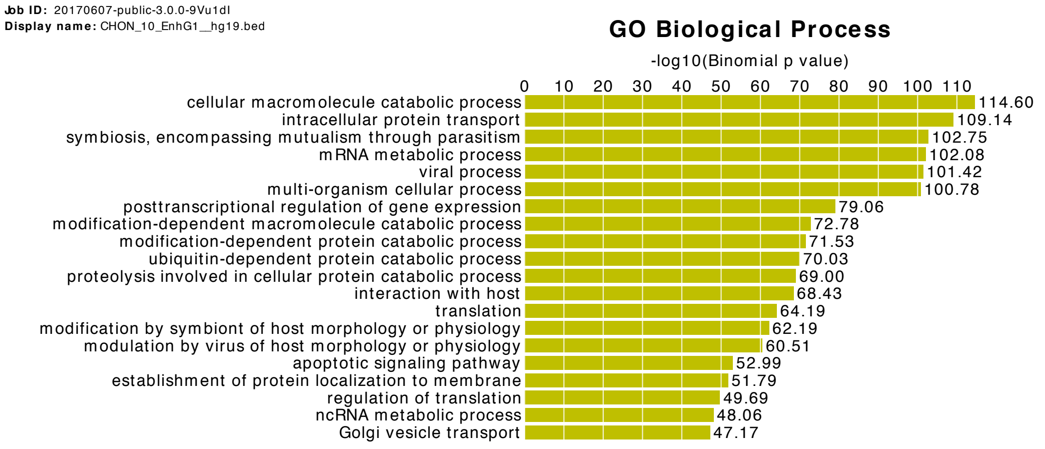

Figure S12 - GREAT GO terms for chondrocyte chromatin state 10_EnhG1

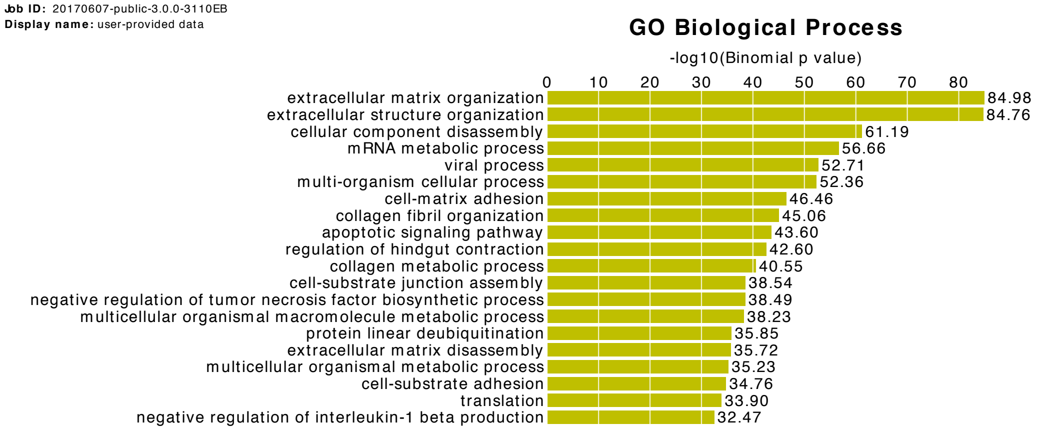

Figure S13 - GREAT GO terms for chondrocyte chromatin state 11_EnhG1

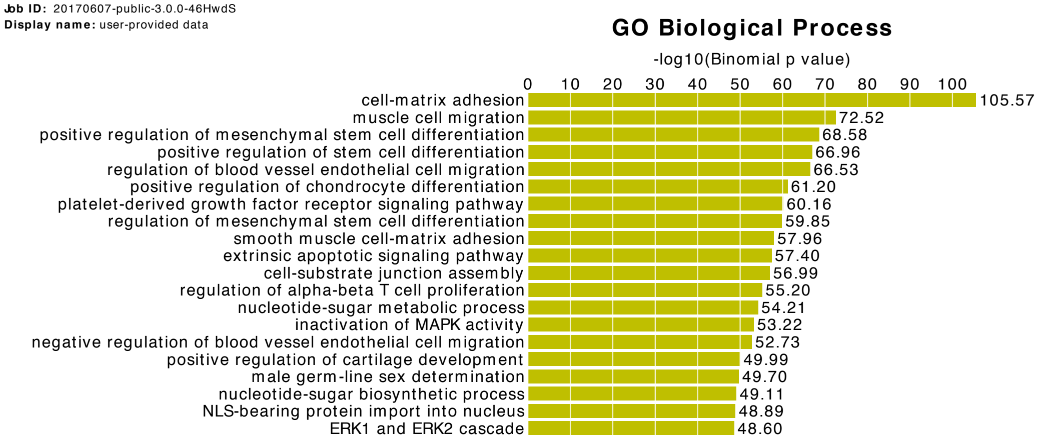

Figure S14 - GREAT GO terms for chondrocyte chromatin state 12_EnhA

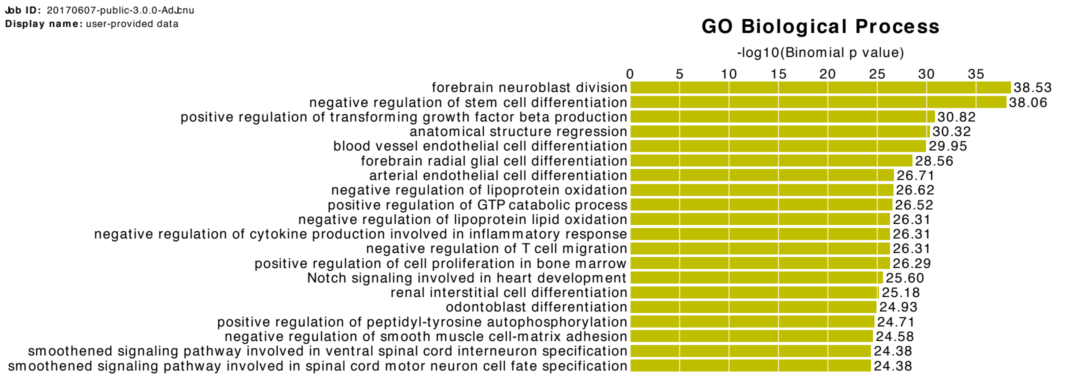

Figure S15 - GREAT GO terms for chondrocyte chromatin state 14_EnhP

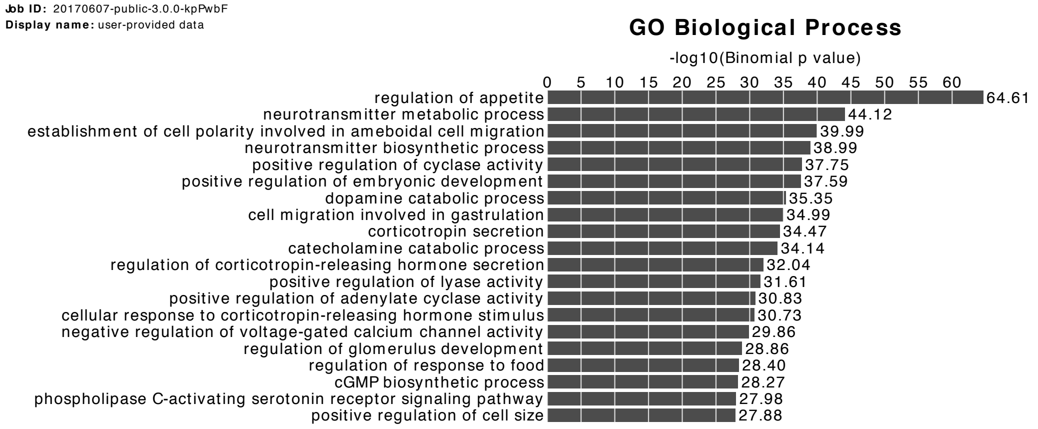

Figure S16 - GREAT GO terms for chondrocyte chromatin state 15_Repr

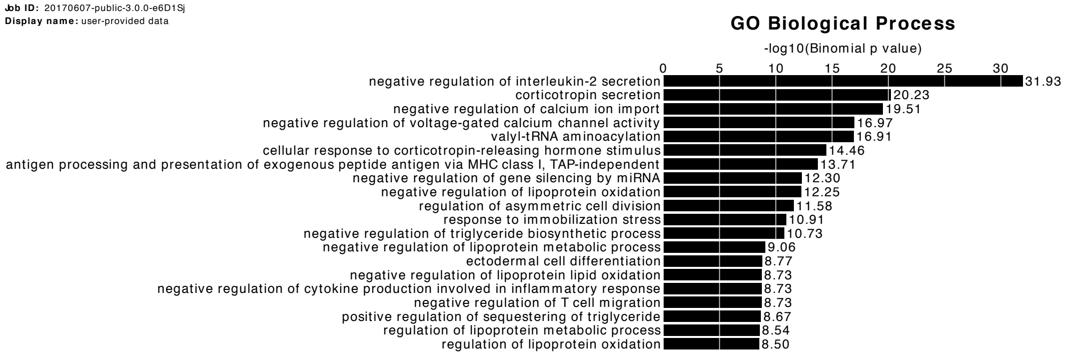

Figure S17 - GREAT GO terms for chondrocyte chromatin state 16_Quies

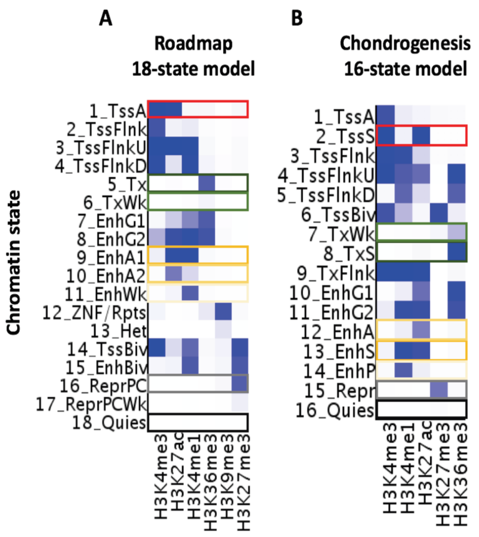

Figure S18 - ChromHMM emission parameters for Roadmap's 18 state model (A) and (B) chondrogenesis 16 state model. Roadmap's chondrogenesis 16 state model. Roadmap's 1_TssA state and the chondrogenesis 2_TssS state comprises equal probabilities of H3K4me3 and H3K27ac histone marks. Likewise, Roadmap's 9_EnhA1 and chondrogenesis 13_EnhS have similar levels of H3K4me1 and H3K27ac.Other states considered comparable were Roadmap's 5_Tx with chondrogenesis 8_TxS, Roadmap's 6_TxWk with chondrogenesis 7_TxWk, Roadmap's 11_EnhWk with chondrogenesis 14_EnhP, Roadmap's 16_ReprPC with chondrogenesis 15_Repr and Roadmap's 18_Quies with chondrogenesis 16_Quies

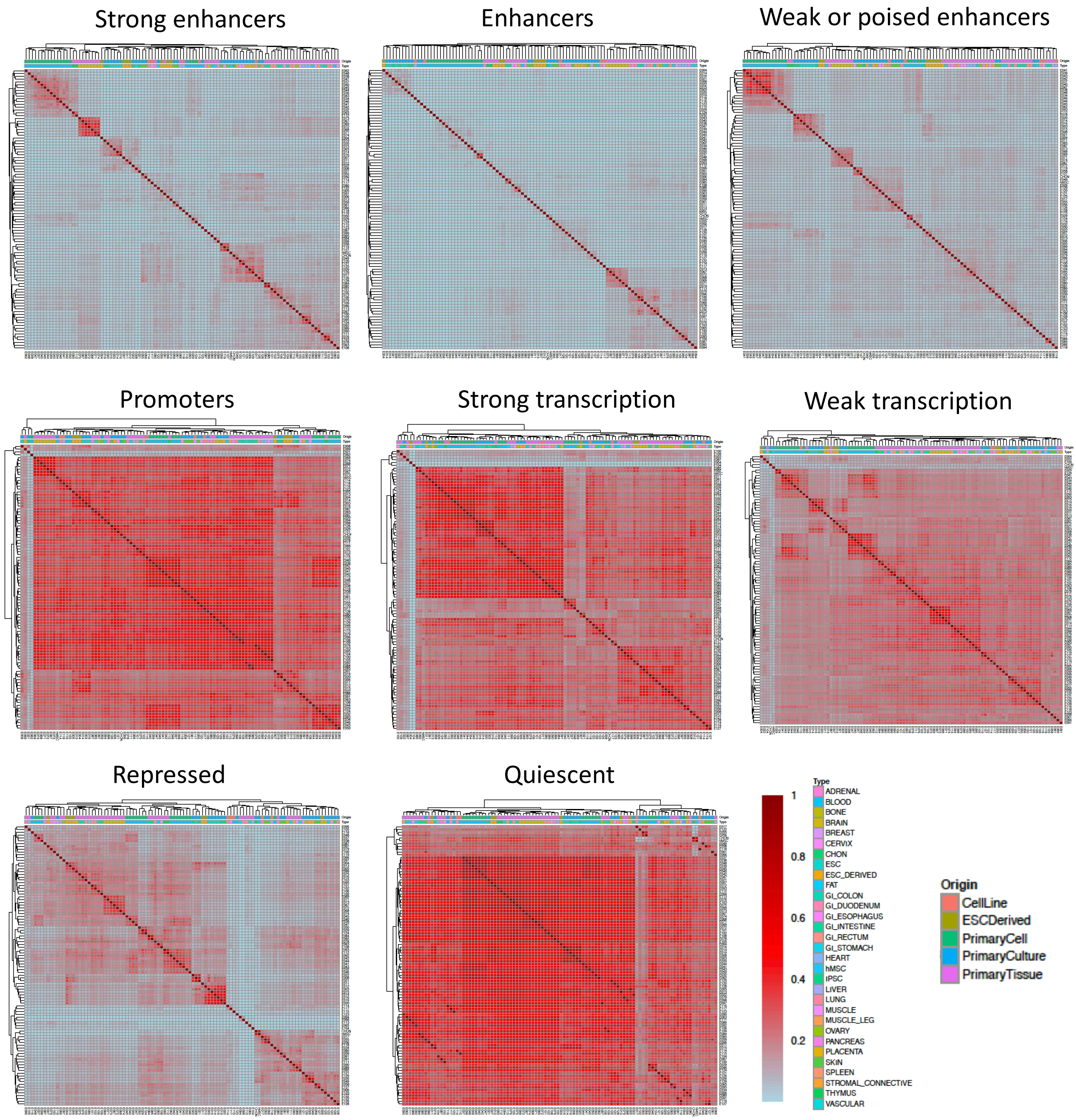

Figure S19 – Heatmaps of Jaccard similarity of cell types between equivalent chromatin states generated in this study and the Roadmap Epigenomics project. The Jaccard similarity metric ranges from 1 (identical overlap) to 0 (no overlap).

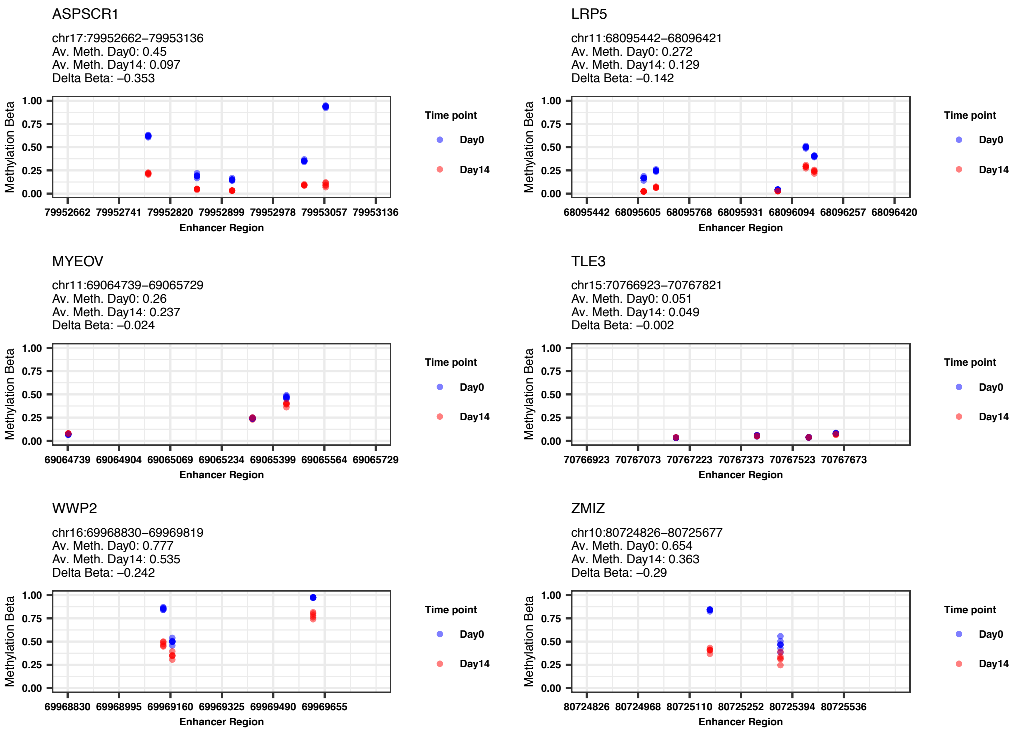

Figure S20 – Methylation in the six enhancer regions selected for luciferase reporter assays. Methylation beta values are shown on the y-axis and the CpG location within the enhancer is shown on the x-axis.

Logo	Database	ID	Alt ID	*p*-value	*E*-value	TP Thresh	TP (%)	FP (%)
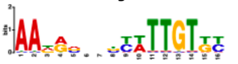	motifs	3	AADRVNNBYWTTGTKY	3.26e-30	3.58e-29	1.07	6504 (10.6%)	8405 (8.7%)
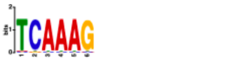	motifs	11	TCAAAG	6.19e-17	6.81e-16	5.73	4838 (7.9%)	6369 (6.6%)
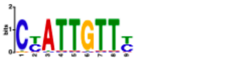	motifs	10	CYATTGTTY	7.75e-17	8.52e-16	1.06	11681 (19.0%)	16498 (17.2%)
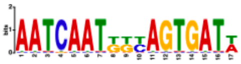	motifs	7	AATCAATKKYAGTGATW	4.35e-4	4.78e-3	3.21	600 (1.0%)	711 (0.7%)
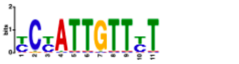	motifs	9	YCYATTGTTYT	1.18e-3	1.30e-2	1.08	3607 (5.9%)	5063 (5.3%)
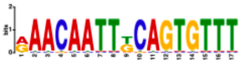	motifs	6	RAACAATTKCAGTGTTT	4.07e-1	4.48e0	23.80	171 (0.3%)	189 (0.2%)
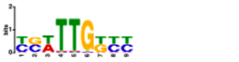	motifs	2	YSWTTGKYY	5.73e-1	6.31e0	3.28	7952 (13.0%)	11794 (12.3%)
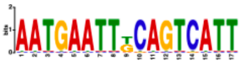	motifs	8	AATGAATTKCAGTCATT	6.88e-1	7.56e0	33.18	137 (0.2%)	149 (0.2%)
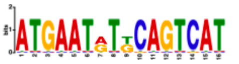	motifs	5	ATGAATRTKCAGTCAT	9.89e-1	1.09e1	3.78	556 (0.9%)	747 (0.8%)
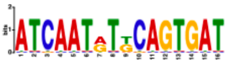	motifs	4	ATCAATRTKCAGTGAT	1.00e0	1.10e1	3.78	433 (0.7%)	643 (0.7%)
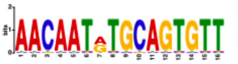	motifs	1	AACAATRTGCAGTGTT	1.00e0	1.10e1	14.43	224 (0.4%)	308 (0.3%)

Figure S21 – Relative enrichment of SOX9 binding motifs in new enhancers to constant enhancers
